# Obesity: systemic and pulmonary complications, biochemical abnormalities, and impairment of lung function

**DOI:** 10.1186/s40248-016-0066-z

**Published:** 2016-07-12

**Authors:** Thiago Thomaz Mafort, Rogério Rufino, Cláudia Henrique Costa, Agnaldo José Lopes

**Affiliations:** Laboratory of Respiration Physiology, Pulmonary Medicine Department, Pedro Ernesto University Hospital, State University of Rio de Janeiro, Boulevard 28 de Setembro, 77, Vila Isabel, 20551-030 Rio de Janeiro Brazil; Postgraduate Programme in Medical Sciences, State University of Rio de Janeiro, Av. Prof. Manoel de Abreu, 444, Vila Isabel, 20550-170 Rio de Janeiro Brazil

**Keywords:** Obesity, Metabolic syndrome, Lung function

## Abstract

Obesity is currently one of the major epidemics of this millennium and affects individuals throughout the world. It causes multiple systemic complications, some of which result in severe impairment of organs and tissues. These complications involve mechanical changes caused by the accumulation of adipose tissue and the numerous cytokines produced by adipocytes. Obesity also significantly interferes with respiratory function by decreasing lung volume, particularly the expiratory reserve volume and functional residual capacity. Because of the ineffectiveness of the respiratory muscles, strength and resistance may be reduced. All these factors lead to inspiratory overload, which increases respiratory effort, oxygen consumption, and respiratory energy expenditure. It is noteworthy that patterns of body fat distribution significantly influence the function of the respiratory system, likely via the direct mechanical effect of fat accumulation in the chest and abdominal regions. Weight loss caused by various types of treatment, including low-calorie diet, intragastric balloon, and bariatric surgery, significantly improves lung function and metabolic syndrome and reduces body mass index. Despite advances in the knowledge of pulmonary and systemic complications associated with obesity, longitudinal randomized studies are needed to assess the impact of weight loss on metabolic syndrome and lung function.

## Background

In recent decades, the prevalence of obesity has increased dramatically, and it has become the most common metabolic disease worldwide, leading to a global epidemic [1]. The etiology of obesity is complex and multifactorial and results from the interaction of genes with the environment, lifestyle, and emotional factors. The modern lifestyle is a potent risk factor for obesity. Declining physical activity levels and increased caloric intake are important environmental factors contributing to obesity [2, 3].

The increasing number of overweight and obese individuals is a serious public health problem that has implications for society and healthcare systems on a global scale. The economic consequences of obesity and associated diseases are not limited to high medical costs but also include indirect or social costs such as decreased quality of life, problems in social adjustment, lost productivity, disability associated with early retirement, and death [4].

Several systemic complications are associated with obesity, some of which lead to severe impairment of organs and tissues. These complications involve mechanical changes caused by the accumulation of adipose tissue and the numerous cytokines produced by adipocytes [5]. The effects of obesity on the respiratory system have been increasingly studied. The accumulation of fat in the body causes changes in respiratory physiology, with consequent impairment of various lung function parameters. Different patterns of body fat distribution differentially and negatively affect the function of the respiratory system [6].

## Review

### Systemic complications related to obesity

Although the relationship between obesity and pulmonary dysfunction is becoming increasingly clear, there is still much controversy regarding whether it occurs in other populations. Complex diseases such as asthma, obstructive sleep apnea (OSA), and chronic obstructive pulmonary disease (COPD) are multifactorial diseases that involve interactions among environmental, genetic, and behavioral factors [7]. Obesity is associated with various diseases and is an important cardiovascular risk factor. Overweight status promotes metabolic and structural changes that increase susceptibility to various diseases, including cardiovascular diseases, metabolic disorders, renal and biliary diseases, and certain types of cancer [7].

Obese individuals exhibit a persistent proinflammatory state that leads to insulin resistance, endothelial dysfunction, systemic arterial hypertension (SAH), and dyslipidemia. These factors culminate in type 2 diabetes mellitus (T2DM) and promote atherogenesis, which in turn increases the risk of coronary heart disease, stroke, and heart failure [8, 9].

Several studies have shown a direct correlation between obesity and OSA. However, the exact pathophysiology of OSA in obese patients remains poorly understood [10]. The mechanisms involved include an increase in neck circumference as well as the direct action of adipose tissue on the airways via a decrease in the luminal diameter of the airway and an increase in the probability of airway collapse [11, 12].

Asthma is also correlated with obesity. Obese individuals with asthma are more likely to have difficult-to-control complications and diseases, and individuals with a higher body mass index (BMI) have a greater risk of developing asthma. The mechanisms involved in this association include increased bronchial hyperresponsiveness (BHR), functional respiratory decline with decreased respiratory volume and flow, chronic systemic inflammation triggered by increased levels of inflammatory cytokines and chemokines, and factors derived from adipocytes, including leptin, adiponectin, and plasminogen activator inhibitor [13, 14]. Several other factors also appear to contribute to the increased risk of asthma in obese individuals, including changes in respiratory function, low exercise tolerance, and predisposition to gastroesophageal reflux [15, 16].

A possible relationship between obesity and COPD has been discussed. An epidemiological study of 650,000 patients revealed that the prevalence of obesity was significantly higher in patients with COPD than in those without COPD (24.6 and 17.1 %, respectively, *p* < 0.0001) [17]. Because pro-inflammatory mediators are present in both obese individuals and in individuals with COPD, these mediators may be the connection between these two conditions [18].

More than one-fifth of the population of the United States and approximately 60 % of obese individuals have metabolic syndrome [19]. In this context, an association between metabolic syndrome and pulmonary disease has been widely debated in recent years [20, 21]. Metabolic syndrome has been identified as an independent risk factor for worsening respiratory symptoms, impairment of lung function, asthma, and pulmonary hypertension. Several possible mechanisms have been proposed to explain these associations, including exposure to high insulin levels during fetal maturation (which induces alterations in airway smooth muscle), the effects of abdominal adiposity, deregulation of adipokine metabolism, and inflammation induced by fat in the lungs [22].

In the gastrointestinal tract, obesity is associated with gastroesophageal reflux disease, cholelithiasis, and liver steatosis. In the osteoarticular system, obesity is correlated with an increased prevalence of osteoarthritis, and in the reproductive system, obesity is correlated with female infertility, polycystic ovary syndrome, and erectile dysfunction [23].

Obesity is also associated with cancers of the breast, cervix, colon, endometrium, esophagus, kidney, liver, ovaries, prostate, and rectum. Furthermore, obese women have a higher prevalence of depression, menorrhagia, amenorrhea, and urinary incontinence. During pregnancy, obesity is associated with a higher risk of maternal complications; obese patients also have a higher incidence of adverse fetal outcomes [23].

### Biochemical and molecular changes

Obesity is associated with a state of chronic systemic inflammation that is driven predominantly by the action of substances released by adipose tissue. Chronic inflammation is caused by activation of the innate immune system, which promotes a pro-inflammatory state and oxidative stress (OS) and a consequent systemic acute-phase response [5]. Systemic inflammation may play a crucial role in the pathogenesis of various obesity-related complications, including metabolic syndrome, T2DM, cardiac disease, liver dysfunction, and cancer.

Adipose tissue is an endocrine and energy storage organ composed of adipocytes, fibroblasts, endothelial cells, and immune cells. These cells secrete hormones and cytokines (adipokines) that exert endocrine, paracrine, and autocrine functions. Under physiological and pathological conditions, adipokines induce the production of reactive oxygen species (ROS), which trigger OS; this, in turn, leads to increased production of other adipokines. During this process, immune cells produce free oxygen radicals that promote a systemic proinflammatory state [24].

Excess adipose tissue is associated with the production of various proinflammatory cytokines, including tumor necrosis factor-α (TNF-α), interleukin-1-β (IL-1β), and interleukin-6 (IL-6) [25]. TNF-α plays a critical role in the inflammatory response of the immune system as well as in the apoptosis of adipose cells, lipid metabolism, hepatic lipogenesis, and the induction of OS. Increased levels of TNF-α promote a response via the release of IL-6, another proinflammatory molecule, and the reduction of levels of anti-inflammatory cytokines such as adiponectin. TNF-α also increases the interaction of electrons with oxygen, generating superoxide anions. TNF-α levels are elevated in obese individuals and decrease with weight loss [26].

IL-1β is a pyrogenic cytokine that is released primarily by monocytes in response to tissue damage or infection. It has recently been proposed that IL-1β is also associated with the proinflammatory response in obesity via the increased production of other cytokines, including IL-6 [27]. IL-6 is secreted by adipocytes, endothelial cells, pancreatic cells, macrophages, and monocytes and participates in the regulation of energy homeostasis and inflammation. IL-6 influences the transition from acute to chronic inflammation by stimulating the synthesis of pro-inflammatory cytokines and the down-regulation of anti-inflammatory targets [28]. Visceral adipose tissue secretes two or three times more IL-6 than subcutaneous adipose tissue via the production of other pro-inflammatory molecules [29]. In humans, high levels of IL-6 are associated with glucose intolerance, T2DM, SAH, and especially obesity. This cytokine may also suppress the activity of lipoprotein lipase and modulate central appetite control at the hypothalamic level [30].

Obese individuals are also more susceptible to oxidative damage. The accumulation of adipose tissue, particularly visceral adipose tissue, induces the synthesis of proinflammatory cytokines, including TNF-α, IL-1, and IL-6. These cytokines promote the generation of reactive oxygen and nitrogen species by macrophages and monocytes, which may lead to increased OS [25]. ROS induce the release of pro-inflammatory cytokines and the expression of adhesion molecules, including connective tissue growth factor, insulin-like growth factor I, platelet-derived growth factor, and vascular cell adhesion molecule I, all of which trigger OS and appear to accelerate aging and cell death, with numerous systemic consequences [29, 31].

Another mechanism involved in the increased susceptibility of obese individuals to oxidative damage is the depletion of enzymes that are active in antioxidant pathways, including superoxide dismutase (SOD), glutathione peroxidase, and catalase. Antioxidant pathways associated with vitamins A, C, and E and beta-carotene also seem to be depleted [32]. Compared with normal-weight individuals, SOD activity is significantly decreased in obese subjects [33]. Oxidative damage leads to the increased production of free radicals, OS, mitochondrial DNA damage, and depletion of adenosine triphosphate, culminating in damage to cellular structures. The cellular damage caused by this lipotoxic state is a direct consequence of the cascade of proinflammatory cytokines released by adipose tissues [34].

Adipose tissue is a source of several bioactive adipokines, including leptin, adiponectin, visfatin, resistin, apelin, and type I plasminogen activation inhibitor (PAI-I). These adipokines are directly associated with physiological and pathological processes involving OS [5].

Leptin is a hormone that is secreted by adipocytes in amounts that are directly proportional to adipose tissue mass and triglyceride levels. The function of leptin is primarily anorexigenic; it binds to proteins, circulates in the plasma, reaches the central nervous system, and promotes satiety. However, it has been postulated that obesity is associated with increased levels of leptin and that a decrease in leptin’s anorexigenic effect via resistance mechanisms occurs in obese patients [25]. The mechanism by which leptin promotes OS has not been determined. However, one hypothesis is that hexamethylene bis-acetamide inducible-1 (Hexim1) is involved in maintaining whole-body energy balance [35]. These hormones may act by inducing the synthesis of cytokines such as TNF-α, interleukin-2 (IL-2), and interferon-γ and can exert their functions in various cell types, including T cells, monocytes, neutrophils, and endothelial cells [36]. Studies have also shown that leptin increases serum levels of C-reactive protein (CRP), confirming its pro-inflammatory effect [36].

In contrast to leptin, adiponectin, which is secreted by differentiated adipocytes, has anti-inflammatory and anti-atherogenic effects. It inhibits the adhesion of monocytes to endothelial cells, the transformation of macrophages into foam cells, and the activation of endothelial cells [37]. Adiponectin also decreases TNF-α and CRP levels and increases the release of nitric oxide (NO) from endothelial cells. A deficiency in this hormone results in decreased levels of NO and reduced leukocyte adhesion, leading to chronic vascular inflammation [38]. It has also been observed that TNF-α and IL-6 are potent inhibitors of the synthesis of adiponectin and other adipokines, including visfatin. Exposure of adipocytes to high levels of ROS also suppresses the production of adiponectin. These mechanisms explain why low levels of adiponectin are found in obese individuals [39].

Visfatin, a recently discovered adipokine, has been positively correlated with the accumulation of adipose tissue. In addition, the level of this hormone decreases with weight loss [40]. Visfatin has pro-oxidant and pro-inflammatory activity and is elevated in obese individuals compared with normal-weight individuals [41]. It stimulates leukocytes and the production of pro-inflammatory cytokines (IL-1, IL-6, and TNF-α) and promotes the generation of ROS [41].

Resistin, a compound present at low levels in adipocytes and at high levels in circulating monocytes, was initially described as an adipokine that is involved in the regulation of appetite, energy balance, and insulin resistance. However, other studies have shown that resistin is associated with an increase in the incidence of cardiovascular disease in obese individuals. The mechanisms involved are directly related to OS and involve the activation of endothelial cells and the upregulation of adhesion molecules and pro-inflammatory cytokines in vascular walls [42].

In summary, the dysfunction of adipose tissue can induce systemic OS and lead to abnormal production of adipokines, which contributes to the development of obesity-related disorders. Furthermore, the level of oxidative damage biomarkers is increased in obese individuals and is directly correlated with BMI, percentage of body fat, and levels of triglycerides and low-density lipoproteins [43]. The accumulation of fat, particularly abdominal visceral fat, impairs antioxidant mechanisms [44]. All these events lead to a chronic and persistent proinflammatory state that results in systemic pathologies.

### Obesity and lung function

**Fig. 1:**
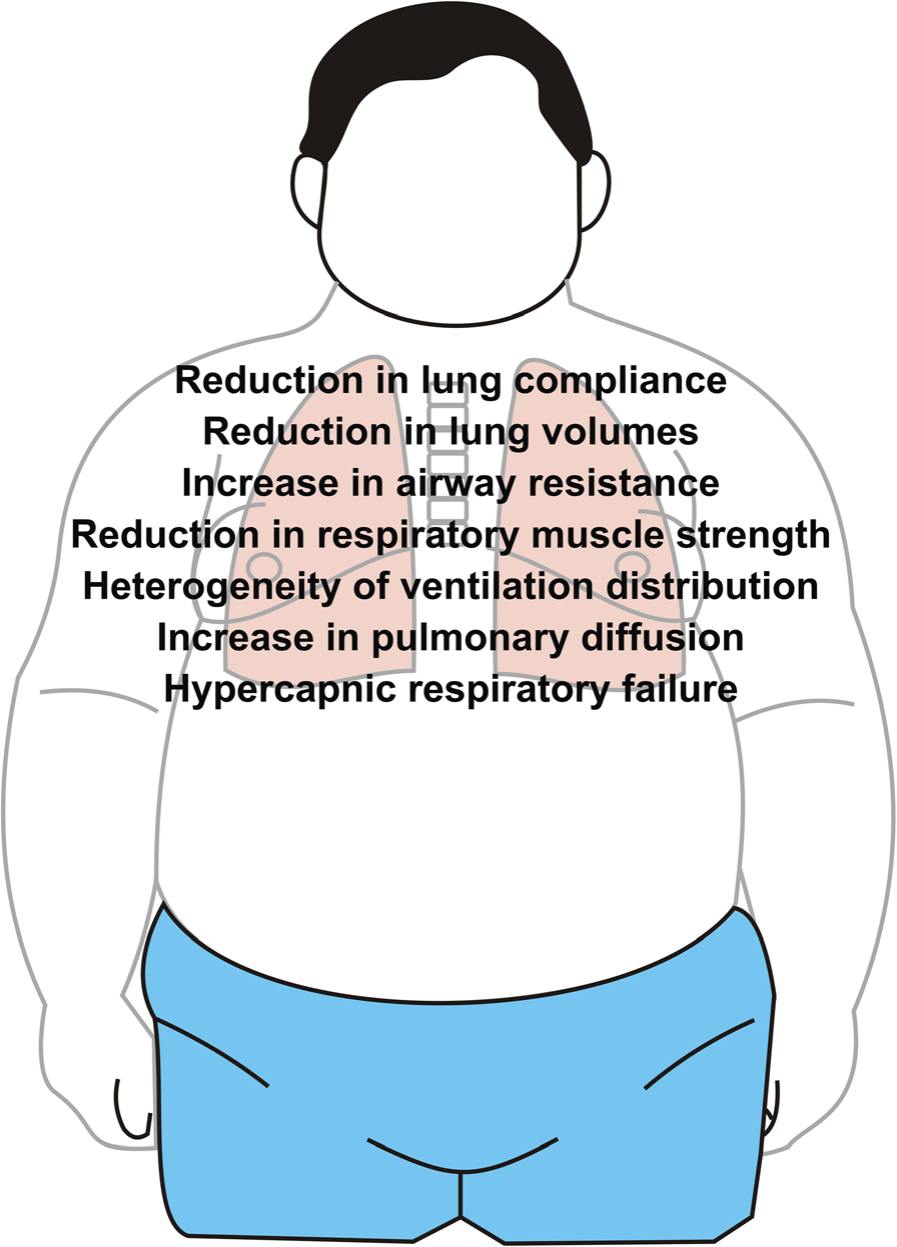
Pulmonary function abnormalities resulting from obesity

Obesity affects the respiratory system by several mechanisms, including direct mechanical changes due to fat deposition on the chest wall, abdomen, and upper airway as well as systemic inflammation [45]. It increases the work of breathing and, therefore, increases neural respiratory drive, in addition to causing respiratory sleep disorders and eventually hypercapnic respiratory failure [46]. In this context, tests of pulmonary function may be useful in evaluating whether a physiological change can be explained by the well-known effects of obesity on the respiratory system. Moreover, the detection of changes in the respiratory system resulting from obesity may be important because several of these changes can be reversed by weight loss or by surgical treatment of obesity (Fig. 1).

In normal respiration, the diaphragm contracts, pushing the abdominal contents down and forward. At the same time, the contraction of the external intercostal muscles pulls the ribs upward and forward [47]. In obese individuals, this mechanism is impaired because the excess body fat that lines the chest and occupies the abdomen limits the action of the respiratory muscles. These structural changes in the thoracic-abdominal area restrict diaphragmatic mobility and rib movement, which promotes changes in the dynamics of the respiratory system and reduces its compliance, leading to mechanical impairment of the respiratory muscles [48]. Reduction in lung compliance can also result from increased pulmonary blood volume, closure of dependent airways with the formation of small areas of atelectasis, or increased alveolar surface tension due to a reduction in FRC [45, 49]. In addition, changes in the neural control of breathing and increases in thoracic blood volume due to fat deposition in the chest also promote changes in pulmonary function parameters [50]. Of note, Rasslan et al. [51] observed that adipose tissue is an endocrine and paracrine organ that produces many cytokines and bioactive mediators, resulting in a pro-inflammatory state that may be associated with pulmonary hypoplasia, atopy, BHR, and increased risk of asthma in obese individuals.

#### Lung volume

Evaluation of static lung volume primarily indicates a reduction in the expiratory reserve volume (ERV), functional residual capacity (FRC), and total lung capacity (TLC). Reductions in FRC and ERV are detectable even at a modest increase in weight. This results from a shift in the balance of inflationary and deflationary pressures on the lung due to the mass load of adipose tissue around the rib cage and abdomen [49]. Elevated intra-abdominal pressure can be transmitted to the chest. This dramatically reduces the FRC and ERV and requires patients to breathe in a less efficient part of their pressure-volume curve, which in turn increases the work of breathing [46].

Jones and Nzekwu [52] reported that decreases in ERV, FRC, and TLC seem to exhibit an exponential correlation with increased BMI and are directly correlated with the mechanical effects produced by fat deposition in the chest and abdomen. According to this study, obesity decreases respiratory system compliance and creates mechanical restraints on the muscles responsible for breathing. In addition, Mafort et al. [53] used spirometry and whole body plethysmography to evaluate 30 patients who were overweight or obese and showed that the primary change in lung volume in these patients was reduced ERV. According to these authors, deposition of fat in the thoracic-abdominal region is one of the main causes of the observed reduction in ERV. It is noteworthy that marked reductions in ERV may lead to abnormalities in ventilation distribution, with closure of airways in the dependent zones of the lung and inequalities in the ventilation-perfusion ratio [49].

#### Airway function

Despite an association with increased BMI, airway function as measured by spirometry is little affected by obesity except in morbidly obese individuals [45]. However, the use of spirometry to evaluate lung function in morbidly obese subjects revealed a proportional reduction in forced vital capacity (FVC) and forced expiratory volume in one second (FEV_1_), suggesting the occurrence of restrictive lung disease [54, 55]. The reduction in FEV_1_ and FVC appears to be directly associated with the degree of obesity in morbidly obese subjects with more severe restrictions. However, obesity has little direct effect on airway caliber. The FEV_1_/FVC ratio is generally well preserved or elevated even in morbidly obese individuals, indicating that FEV_1_ and FVC are affected at the same rate [56]. A reduction in expiratory flows in an obese individual is unlikely to indicate bronchial obstruction unless the flow measurements have been normalized for the reduction in FVC [49].

Whole-body plethysmography, impulse oscillometry, or the forced oscillation technique (FOT) can also be used to assess the mechanical properties of the airways in obese individuals, more precisely through the measurement of airway resistance (Raw) [57]. Given that Raw is highly dependent on lung volume and hence is affected by any reduction in the FRC, Raw is increased in obese individuals. In contrast, specific airway resistance, which is corrected for lung volume, is within the normal range in such individuals. However, some studies have suggested that the increase in Raw is not completely explained by reduced lung volume because differences between obese individuals and non-obese individuals may persist after correction of Raw for lung volume [57, 58]. The cause of the increase in Raw is unknown; one possibility is that the structure of the airway may be remodelled by exposure to proinflammatory adipokines or by lipid deposition [49].

#### Respiratory muscle strength

The function of the respiratory muscles may be impaired with increasing obesity, possibly due to the load imposed on the diaphragm. The observed dysfunction of the respiratory muscles can be partially explained by the increased resistance imposed by the presence of excess fatty tissue on the chest and abdomen, which causes mechanical disadvantage to these muscles [59].

Respiratory muscle strength can be assessed by measuring maximal inspiratory pressure (MIP) and maximal expiratory pressure (MEP). In obese individuals, both MIP and MEP may be reduced. The impairment of respiratory muscles is multifactorial; although some studies indicate that the diaphragm exhibits higher electromyographic activity in obese individuals than in normal-weight individuals, ineffective muscle contraction and premature fatigue also occur [50, 60, 61], indicating that the reduction in MIP and MEP may be due to distension of the diaphragmatic muscles, increased respiratory effort, and ineffective muscle biomechanics caused by fat deposition in the thoracic and abdominal regions. Moreover, when in the supine position, the weight of the abdomen in obese individuals causes the diaphragm to ascend into the chest, resulting in the closure of small airways at the base of the lung and thereby generating an intrinsic positive end-expiratory pressure that results in increased ventilatory work and consequent muscle impairment [60, 61].

#### Ventilation distribution and gas exchange

Most obese individuals present an arterial partial pressure of oxygen (PaO_2_) within the normal range. However, among morbidly obese subjects, the alveolar-arterial oxygen gradient [P(A-a)O_2_] is slightly widened because of the presence of areas of atelectasis and maldistribution of ventilation, which can cause a major ventilation-perfusion imbalance. In these individuals, the lower parts of the lungs are relatively poorly ventilated and perfused, possibly due to the closure of small airways, whereas the upper regions of the lungs exhibit enhanced ventilation [45, 62].

When assessing the carbon monoxide lung diffusion capacity (DLco), it should be noted that lung tissue perfusion is a determining factor because most perfused areas have higher concentrations of red blood cells and consequently higher diffusion of this gas than non-perfused areas. This factor is important when evaluating the diffusion of gases in obese individuals. Fat deposition in the thoracic region leads to higher vascularization in this area. This explains, at least in part, the increase in the DLco observed in the obese population [63]. In a recent study, the authors observed elevated DLco in 23.3 % of obese individuals, and elevated DLco was most frequent in individuals with the highest accumulation of fat in the thoracic region (*r*_*s*_ = 0.42; *p* < 0.01) [64].

### Relationships between obesity, asthma, and bronchial hyperresponsiveness

Obesity has been associated with a higher incidence, prevalence, and severity of asthma and with altered pulmonary function, poor treatment response, and high morbidity [15, 65–68]. The incidence of asthma is 1.47 times higher in obese individuals than in non-obese individuals, and a three-unit increase in BMI is associated with a 35 % increase in the risk of asthma [69, 70]. Decreases in FRC and tidal volume in addition to sedentary lifestyle and limited ability to perform+ physical activities among obese individuals may worsen asthma symptoms [15, 71, 72]. In a cohort study of more than 25,000 children and adults with asthma, Schatz et al. [73] showed that a higher BMI was associated with worsened asthma control and an increased risk of asthma exacerbations.

The inflammatory changes described in obese individuals have been cited as factors that might affect the clinical manifestations of asthma in these individuals. The inflammatory condition of an obese individual, which includes higher expression levels of leptin, adiponectin, TNF-α, transforming growth factor-β (TGF-β), CRP, and eotaxin, determines how these inflammatory mechanisms overlap with those involved in asthma and may exacerbate the influence of these cytokines on the contractility of the muscles of the airways [68, 74].

By reducing functional lung volume, obesity can change airway diameter due to the interdependence of the airway and the adjacent pulmonary parenchyma; these effects favor the development of BHR even in non-asthmatic individuals. BHR has the potential to enhance the effects of obesity on airway closure and hence on the distribution of ventilation [49]. Torchio et al. [68] evaluated 41 healthy subjects through the methacholine challenge test measured in a body plethysmograph and FOT and demonstrated that BHR was significantly associated with obesity. These authors also observed that in obese men, but not in obese women, BHR was associated with a decrease in lung volume. However, it remains unclear whether conditions associated with BHR, such as obesity, are a risk factor for asthma. Studies have provided conflicting results. Confounding factors include the different mechanisms involved in obesity and asthma, self-reported diagnosis of asthma, gender differences, the absence of synergistic effects of obesity and asthma on lung function, and the use of different methods to measure lung function [75–77].

Although the association between asthma and obesity remained uncertain until recently, the existence of different asthma phenotypes is now well recognized. More recently, Bates [78] highlighted two phenotypes of asthma in obese individuals: an early-onset allergic (EOA) form that is complicated by obesity and a late-onset non-allergic (LONA) form that occurs only in the setting of obesity. Whereas obese LONA asthmatics have more compliant airways, obese EOA asthmatics display considerable inflammatory thickening of the airways. Thus, these two phenotypes appear to be quite distinct pathological conditions in obese individuals with asthma.

### Patterns of body fat distribution and pulmonary function

Accumulation of fat in the thoracic and abdominal regions is likely to directly affect the downward movement of the diaphragm and chest wall properties [49]. The pattern of body fat distribution seems to be relevant to the changes in lung function observed in overweight and obese individuals. Changes in chest wall compliance are more affected by the amount of fat in both the chest and upper abdomen than by the amount of fat only in the chest, suggesting that respiratory system mechanics may differ in obese individuals with the same BMI but with different patterns of body fat distribution [45]. The pattern of body fat distribution can be assessed using several strategies, including anthropometric methods, electrical bioimpedance, and dual-energy X-ray absorptiometry (DXA) [79–81]. Bioelectrical impedance analysis (BIA) has been widely used due to its high speed of information processing and because it is a non-invasive, convenient, and relatively inexpensive method that estimates the distribution of fluids in the intra- and extracellular spaces in addition to the body components [82]. DXA is a noninvasive method and is considered the gold standard for body composition assessment. It uses low-dose x-rays and permits the assessment of both total body fat and fat in various body compartments, including the thoracic, android, and gynoid regions [64, 83].

Obesity is associated with reduced respiratory system compliance, which itself is exponentially correlated with BMI, waist circumference, and waist-hip ratio [45]. Nevertheless, lung volumes are only slightly associated with BMI, whereas DXA-derived variables present highly significant correlations with FRC and ERV in both men and women [49]. The android pattern of body fat deposition appears to negatively influence lung volume and lung capacity by generating increased resistance to diaphragmatic contraction and impairing respiratory mechanics. This would also explain the greater loss in FEV_1_ and FVC in obese men than in women with a corresponding BMI because the gynoid pattern prevails in women [84, 85]. In a recent study, Mafort et al. found a significant correlation between TLC and waist circumference (*r*_*s*_ = −0.34; *p* = 0.03). These results reinforce the idea that abdominal fat plays a role in the development of restrictive lung disease and its deleterious effect on mechanical ventilation [64].

### Dyspnea on exertion in obese individuals

Dyspnea on exertion is a common complaint of obese adults. However, the mechanism responsible for this symptom is not well defined yet [86]. Almost 40 % of obese individuals complain of dyspnea on exertion, an incidence that is higher than that in general population [87]. Obesity has clear potential to directly affect respiration during exercise because there is an increase in oxygen consumption (VO_2_) and carbon dioxide production (VCO_2_) due to stiffening of the respiratory system with the increase in mechanical work needed to sustain exercise. Thus, even a slight increase in minute ventilation (V_E_) relative to resting levels can result in a considerable increase in the ratio between VO_2_ and respiratory work in obese adults. This ratio increases considerably in conditions in which higher levels of V_E_ are required, as during exercise, and this may result in dyspnea on exertion [87–89].

Cardiopulmonary exercise testing (CPX) can provide valuable information on the performance of the cardiac and respiratory systems in obese individuals with dyspnea on exertion. Using CPX, Bernhardt et al. [87] compared obese men with dyspnea grade ≤2 with those with dyspnea grade ≥4 assessed by the Borg scale. In that study, the authors found no association between the level of perception of dyspnea and VO_2_ and concluded that differences in the intensity of exercise, ventilatory demand, cardiovascular fitness, or quality of the respiratory sensation do not seem to play an important role in the development of dyspnea on exertion in these individuals. Also using CPX, Hothi et al. [90] evaluated VO_2max_/kg in 152 obese individuals and 173 non-obese individuals with severe heart failure (HF). They found that VO_2max_/kg is not a reliable indicator of cardiac fitness in all patients. Instead, they found that despite having lower VO_2max_/kg, obese patients with HF are capable of generating higher cardiac power than nonobese patients with HF. These results argue against the widespread use of VO_2max_/kg as a cardiac conditioning indicator for all HF patients.

Interestingly, Carpio et al. [91] evaluated the performance of obese patients with asthma and obese patients with misdiagnosed asthma (the presence of asthma-like symptoms) compared with obese control subjects during CPX. These authors observed that the level of dyspnea and the Borg-VO_2_ slope during CPX were higher in obese patients with asthma and in patients with misdiagnosed asthma than in obese control subjects. These authors concluded that the presence of asthma-like symptoms in obese individuals can be attributed to an increased perception of dyspnea, which, during exercise, is mainly associated with systemic inflammation and excessive ventilation for metabolic demands.

### Relationships between obesity, obesity hypoventilation syndrome, and obstructive sleep apnea

Obesity is the most common known risk factor for the development of OSA [92]. The prevalence of OSA associated with high rates of morbidity and mortality increases with age; the peak incidence occurs at approximately age 55, and the condition is more prevalent in males than in females by a ratio of 2:1 [93]. OSA is a systemic disease that causes an increase in TNF-α, IL-6, insulin resistance, and glucose intolerance; these inflammatory cytokines have also been implicated in the immunological mechanisms of obesity [94]. Central obesity and increased neck circumference are predisposing factors for OSA [95, 96]. The resulting reduction or interruption of airflow, which occurs despite inspiratory effort, causes poor alveolar ventilation and oxyhemoglobin desaturation and, in cases of prolonged events, a progressive increase in the arterial partial pressure of carbon dioxide [97].

There is a strong association between OSA and metabolic syndrome as a whole or with its individual components [20]. The prevalence of metabolic syndrome in patients with OSA is 60 %, significantly higher than in general population [98]. This association is partially explained by the fact that patients with OSA are more likely to have high visceral adiposity as well as abnormal glucose metabolism [20, 99]. There is ample evidence suggesting that OSA may exacerbate or induce the majority of the components of metabolic syndrome. Some of these effects can be improved with the use of continuous positive airway pressure. However, the modest and inconsistent benefits obtained with this technique suggest that factors other than intermittent hypoxia or the apnea-hypopnea index may play an important role [20].

Some obese individuals develop obesity hypoventilation syndrome (OHS), which is defined by the triad of obesity, daytime hypoventilation, and sleep-disordered breathing and represents hypoventilation that occurs in the absence of a neuromuscular, mechanical, or metabolic cause [100]. The prevalence of OHS is estimated to be 8.5 % in patients with OSA and 19-31 % in obese subjects [101, 102]. OHS is more prevalent in women than in men, and postmenopausal women with OSA have a higher prevalence of OHS. This has been attributed to hormonal influences, particularly to the role of progesterone as a respiratory stimulant prior to menopause [103, 104]. Compared with eucapnic obese patients, patients with OHS have severe upper airway obstruction, restrictive pulmonary damage, decreased central respiratory drive, increased incidence of pulmonary hypertension, and increased mortality [100]. Among the possible mechanisms involved in the pathogenesis of OHS, some studies have reported damage to respiratory mechanics caused by obesity, leptin resistance leading to central hypoventilation, respiratory sleep disorders, and impaired compensatory responses to acute hypercapnia [97, 100, 102]. With respect to pulmonary function, patients with OHS present a reduction in chest wall compliance of approximately 2.5-fold compared to patients with eucapnic obesity, as well as increased pulmonary resistance that is likely secondary to the reduction in FRC [100].

### Impact of treatment of obesity and weight loss on lung function

As previously reported, obesity causes a number of changes in pulmonary function parameters. It is also known that weight loss improves these parameters, supporting the hypothesis that respiratory changes caused by obesity are a direct result of excess weight [45].

Several studies have shown that ERV, one of the parameters that is most significantly altered in obese individuals, increases after weight loss, adopting a calorie-restricted diet, or bariatric surgery [105, 106]. Hakala et al. [107] found a considerable increase in the ERV of patients whose BMI decreased from 45 to 39 kg/m^2^ after adopting a calorie-restricted diet. Babb et al. showed that even modest reductions in weight, i.e., a decrease in BMI from 35 to 33 kg/m^2^, induce an increase in end-expiratory lung volume during submaximal exercise [108]. Weight loss also causes changes in other parameters, including FRC, TLC, and gas exchange, resulting in increased blood oxygenation [107]. Respiratory muscle strength and dyspnea also improve after weight loss [109, 110].

In a recent publication, Mafort et al. [64] showed that obese and overweight patients exhibited a significant reduction in BMI after six months of intragastric balloon therapy; the median BMI value decreased from 39.1 kg/m^2^ at the beginning of the evaluation to 34.5 kg/m^2^ at the end of the evaluation (*p* = 0.0001). The reduction in BMI was accompanied by statistically significant reductions in TLC (*p* = 0.0001), FRC (*p* = 0.0001), residual volume (*p* = 0.0005), and ERV (*p* = 0.0001).

In obese patients with asthma, both surgical and nonsurgical weight loss are associated with improvement in symptoms, decreased use of medication, increased effectiveness of drug therapy, and a reduction in risk of exacerbation and hospital admission rate [111]. Improvements in lung function, including FEV_1_, FVC, and Raw, after weight loss in obese patients with asthma have also been reported in several studies [66, 112–114]. In a randomized study of obese adult patients with severe uncontrolled asthma, Dias-Júnior et al. [114] showed that following a weight loss program for a period of 6 months was associated with improvement in asthma control and lung function.

A decrease in BHR to methacholine after weight loss in obese individuals with asthma was reported by Al-Alwan et al. [112] and by van Huisstede et al. [115]. In a prospective controlled study of obese individuals with asthma, Pakhale et al. [66] observed a significant improvement in BHR to methacholine compared to controls (*p* = 0.009) after 3 months of a behavioral weight loss program. Boulet et al. [67] evaluated severely obese patients with asthma before and after bariatric surgery and observed reduced BHR, increased lung volume, and noticeably decreased asthma symptoms and medication required to control asthma 12 months after surgery. This study also showed that reduction in BMI and improved BHR were correlated with a reduction in CRP.

## Conclusions

Obesity causes mechanical compression of the diaphragm, lungs, and chest cavity, which can lead to restrictive pulmonary damage. Furthermore, excess fat decreases total respiratory system compliance, increases pulmonary resistance, and reduces respiratory muscle strength. It is interesting that metabolic syndrome also changes lung function and that the combination of obesity and metabolic syndrome seems to impair lung function even further. In obese and overweight patients, a strong correlation exists between lung function and body fat distribution, with greater impairment when fat accumulates in the chest and abdomen. Despite advances in the knowledge of pulmonary and systemic complications and of the biochemical abnormalities associated with obesity, longitudinal randomized studies are needed to assess the impact of weight loss on metabolic syndrome and lung function.

## Abbreviations

BHR: bronchial hyperresponsiveness
BMI: body mass index
CPX: cardiopulmonary exercise testing
CRP: C-reactive protein
DLco: carbon monoxide lung diffusion capacity
DXA: dual-energy X-ray absorptiometry
EOA: early-onset allergic
ERV: expiratory reserve volume
FEV_1_: forced expiratory volume in one second
FOT: forced oscillation technique
FRC: functional residual capacity
FVC: forced vital capacity
Hexim1: hexamethylene bis-acetamide inducible-1
HF: heart failure
IL-1: interleukin-1
IL-1β: interleukin-1-β
L-6: interleukin-6
LONA: late-onset non-allergic
MEP: maximal expiratory pressure
MIP: maximal inspiratory pressure
NO: nitric oxide
OHS: obesity hypoventilation syndrome
OS: oxidative stress
OSA: obstructive sleep apnea
P(A-a)O_2_: alveolar-arterial oxygen gradient
PAI-I: type I plasminogen activation inhibitor
PaO_2_: arterial partial pressure of oxygen
ROS: reactive oxygen species
SAH: systemic arterial hypertension
SOD: superoxide dismutase
T2DM: type 2 diabetes mellitus
TGF-β: transforming growth factor-β
TLC: and total lung capacity
TNF-α: tumor necrosis factor-α
VCO_2_: carbon dioxide production
V_E_: minute ventilation
VO_2_: oxygen consumption
